# Survival rate variation among different types of hospitalized traumatic cardiac arrest: A retrospective and nationwide study

**DOI:** 10.1097/MD.0000000000011480

**Published:** 2018-07-13

**Authors:** Chung-Yu Lai, Shih-Hung Tsai, Fu-Huang Lin, Hsin Chu, Chih-Hung Ku, Chun-Hsien Wu, Chi-Hsiang Chung, Wu-Chien Chien, Ching-Tsan Tsai, Huan-Ming Hsu, Chi-Ming Chu

**Affiliations:** a Graduate Institute of Medical Sciences, National Defense Medical Center; b Department of Emergency Medicine, Tri-Service General Hospital, National Defense Medical Center; c School of Public Health, National Defense Medical Center; d Graduate Institute of Aerospace and Undersea Medicine, National Defense Medical Center, Taipei City; e Department of Health Industry Management, Kainan University, Taoyuan City; f Division of Cardiology, Tri-Service General Hospital, National Defense Medical Center, Taipei City; g Big Data Research Center, Fu-Jen Catholic University, New Taipei City; h Department of Public Health, China Medical University, Taichung City; i Department of Surgery, Tri-Service General Hospital Songshan Branch, National Defense Medical Center, Taipei City; j Department of Healthcare Administration and Medical Informatics, College of Health Sciences, Kaohsiung Medical University; k Department of Medical Research, Kaohsiung Medical University Hospital, Kaohsiung City, Taiwan.

**Keywords:** ICD-9-CM, intensive care unit, postresuscitation, survival to discharge, traumatic cardiac arrest, ventricular fibrillation

## Abstract

Studies regarding the prognostic factors for survival conditions and the proportions of survival to discharge among different types of hospitalized traumatic cardiac arrest (TCA) during the period of postresuscitation are limited.

This nationwide study was designed to determine certain parameters and clarify the effect of various injuries on the survival of hospitalized TCA patients to discharge.

Data were retrieved from the National Health Insurance Research Database (NHIRD) from 2007 to 2013 in Taiwan. We reviewed patients with a diagnosis of TCA using International Classification of Disease Clinical Modification, 9th revision codes (ICD-9-CM codes). Patients identified for analysis were simultaneously coded in traumatic etiology (ICD-9-CM codes: 800–999) and cardiac arrest (ICD-9-CM codes: 427.41 or 427.5). The determinants and effects of different types of injury on survival were evaluated by SPSS 22.0 (IBM, Armonk, NY).

A total of 3481 cases of hospitalized TCA were selected from the NHIRD. The overall rate of survival to discharge was 22.1%. The results indicated a decreased adjusted odds ratio (aOR) of survival to discharge with higher numbers of organ failure (aOR: 0.82; 95% confidence interval [CI]: 0.73–0.92). Patients with ventricular fibrillation had a better discharge rate (aOR: 4.33; 95% CI: 3.29–5.70). Two parameters, transfer to another hospital and the number of intensive care unit beds, were positively correlated with survival. Compared with traffic accidents, different injuries associated with survival to discharge were identified; the aOR (95% CI) was 1.89 (1.12–3.19) for poisoning, 1.63 (1.13–2.36) for falls, and 2.00 (1.36–2.92) for drowning/suffocation.

This study has shown that hospitalized TCA patients with multiple organ failure may be less likely to be discharged from the hospital. The presence of ventricular fibrillation rhythm on admission increased the odds of survival to discharge. In the phase of postcardiac arrest care, the number of intensive care unit beds and transfer to another hospital were positively correlated with survival. Those events attributed to traffic accidents have a much worse influence on the main outcome.

## Abstract 2

Supplemental Digital Content is available in the text

## 1 Introduction

The chance of survival for patients with cardiac arrest is highly associated with etiology.^[1,2]^ The survival of patients with cardiac arrest originating from medical diseases is approximately 5% to 10%.^[3,4]^ The survival rate of cardiac patients who meet certain conditions and are treated with appropriate resuscitation, such as ventricular fibrillation (VF)/ventricular tachycardia (VT), treatment administered by lay rescuers, or treatment by automatic external defibrillator, can be increased by over 40%.^[5]^ Compared with victims of cardiac arrest with cardiac origins, victims of traumatic cardiac arrest (TCA) generally have a worse outcome. Even with improvements, the rate of survival to discharge for TCA patients tends to be lower (approximately 1–7%).^[6–8]^

Guidelines and algorithms have been published for management of TCA. In 2010, the International Liaison Committee on Resuscitation released guidelines on TCA emergency management, including treatment reversible causes, basic and advanced life support, and emergency intervention.^[3]^ However, resuscitation of TCA patients requires continued improvement. In recent years, postcardiac arrest care has been recognized to improve the survival of patients with cardiac arrest due to medical causes by tightening each component of the chain of survival.^[9]^

As discussed above, the postcardiac arrest care phase is crucial for the prognosis of patients with cardiac arrest due to medical causes. To the best of our knowledge, studies regarding the prognostic factors for survival conditions and the proportions of survival to discharge among different types of hospitalized TCA during the period of postresuscitation are limited. The purpose of the present study was to contextualize the impact of particular parameters on survival to discharge and to elucidate whether different types of injury were associated with survival to discharge among hospitalized TCA patients.

## 2 Methods

### 2.1 Study design and materials

This observational retrospective study was conducted to analyze the outcome of hospitalized TCA patients in Taiwan. Data were obtained from the National Health Insurance Research Database (NHIRD). The National Health Insurance (NHI) Program was initiated in 1995, and the Taiwanese Government enacted the world-famous insurance policy by legislation. NHI, a single-tier medical and health service system managed by the National Health Insurance Administration (NHIA), recruited more than 97% of medical service providers in Taiwan and covered nearly 100% of population.^[10]^

The data we obtained from the NHIRD included anonymous and standardized information obtained from the National Health Research Institute (NHRI) located in Taipei city. The NHIRD, a national representative database, was developed in cooperation with the NHI Program and documents medical diagnoses and treatments according to International Classification of Disease Clinical Modification, 9th revision codes (ICD-9-CM).^[11]^ The NHRI encrypts and scrambles the identification codes of both patients and medical facilities by unidentified processes. Meanwhile, each patient was assigned a nonspecific code and single hospital identifier. Since 2000, the NHRI has also rewritten medical claims and released the NHIRD to academic units or researchers after careful review of application protocols. Ultimately, data from the NHIRD cannot be traced back to individual subjects or facilities to protect privacy. The NHRI annually evaluates the database, including data completeness, quality assurance, and information validity.

The inpatient expenditures by admissions dataset record the detailed information of the inpatient population. Patients enrolled in this study were extracted from that dataset during a 7-year period from 1 January 2007 to 31 December 2013. We analyzed the demographic, clinical, and event characteristics of hospitalized TCA patients. Healthcare system features were collected from a registry for contracted medical facilities and a registry for contracted beds datasets.

### 2.2 Case collection and inclusion criteria

The inpatient expenditures by admissions dataset documented the date of hospital admission, payment, diagnostic/procedural codes, external injury codes, and survival conditions. We reviewed the dataset by diagnostic codes, and cases derived for analysis were simultaneously coded in traumatic etiology (ICD-9-CM codes: 800–999) and cardiac arrest (ICD-9-CM codes: 427.41 or 427.5) from 2007 to 2013. Hospitalized TCA patients with “ICD-9-CM code: 427.41” were specifically defined as VF cardiac rhythm on admission.^[12–16]^ Other inpatient cases were identified as non-VF cardiac rhythm on admission.

During this study period, we identified a large sample comprising 3531 hospitalized TCA patients of all ages. We excluded 50 patients fulfilling one of the following exclusion criteria: no definite outcome (n = 9), transferred to another hospital and lost to follow-up (n = 41). Eventually, 3481 patients were enrolled using statistical processes and classified into survivor and nonsurvivor groups.

### 2.3 Primary outcome and covariate definition

We linked datasets through different calendar years to follow the outcome of patients from admission during the study period. The primary outcome analyzed in this study was the percentage of survival to discharge.

Covariates were divided into the following categories: patient-related factors, healthcare system features, and event parameters. In patient-related factors, the variables included patients’ age, gender, Charlson Comorbidity Index (CCI),^[17,18]^ and organ failures (liver failure [ICD-9-CM codes 570, 572.2, and 573.3], heart failure [ICD-9-CM codes 428, 458.0, 458.8, 458.9, 785.5, 785.51, 785.59, and 796.3], renal failure [ICD-9-CM codes 39.95, 580, 584, and 585], respiratory failure [ICD-9-CM codes 96.7, 518.81, 518.82, 518.85, 786.09, and 799.1], neurological failure [ICD-9-CM codes 89.14, 293, 348.1, 348.3, 780.01, and 780.09], metabolic failure [ICD-9-CM code 276.2], and hematological failure [ICD-9-CM code 286.2, 286.6, 286.9, and 287.3–5]).^[16,19]^ Healthcare system features included the type of admitting hospital (medical center, regional hospital, and local hospital), geographic area (northern, central, southern, and eastern/offshore region), teaching hospital status (yes or no), hospital bed number, and intensive care unit (ICU) bed number. Event parameters contained covariates such as cardiac rhythm on admission (VF or non-VF), transfer to another hospital (yes or no), types of injury (traffic accident, poisoning, fall, drowning/suffocation, homicide/suicide, and others), and calendar year (2007–2010 and 2011–2013).

### 2.4 Data analysis

In descriptive analysis, the percentage is presented to report the distribution of hospitalized TCA patients. In the analytic process, we assessed differences in patient-related factors, healthcare system features, and event parameters between the nonsurvivor and survivor groups. The chi-squared test was used for categorical variables. Variables that reached statistical significance with a 2-tailed *P* value < .10 in univariate tests were included in multivariate logistic regression.

A multiple regression model with odds ratios (ORs) and 95% confidence intervals (CIs) was used to identify factors influencing outcome and calculate the possibility of survival with different types of injury. All covariates in the regression model were considered significant by the enter method. A *P* value < .05 was regarded as the level of significance for 2-tailed tests. Statistical analysis was performed by SPSS 22.0 for Windows (IBM, Armonk, NY).

### 2.5 Ethics approval

Ethics approval was received from the ethical committee of the Institutional Review Board of Tri-Service General Hospital in Taipei, Taiwan (TSGHIRB No. 1-105-05-136). Data collection and protocols regarding hospitalized TCA patients in this study were classified as low risk, and the study was exempt from written informed consent.

## 3 Results

During the 7-year study survey period, we selected 3481 hospitalized TCA patients from the NHIRD in Taiwan. Of these patients, 768 (22.1%) survived to discharge (Table 1). More than 70% of patients were over 45 years old, and two-thirds were male. We examined this dataset to report the prevalence of comorbidity by CCI scores; survivors had higher scores (CCI ≥ 1) than nonsurvivors (37.5% compared with 30.9%, respectively, *P* = .001). Patients with survival to discharge were less likely to exhibit symptoms of organ failure. However, there were no significant differences in age and gender between nonsurvivors and survivors.

**Table 1:**
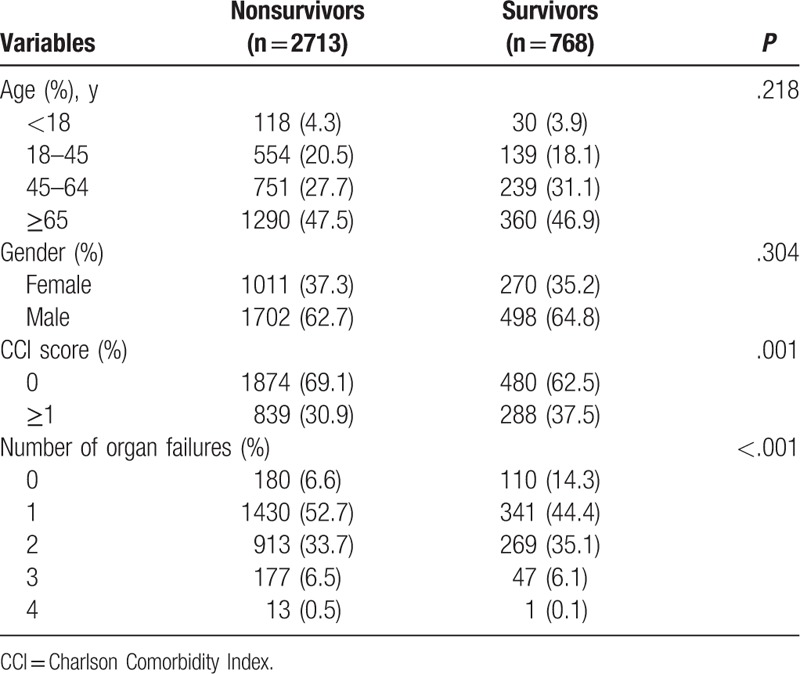
Characteristics of patient-related factors among hospitalized traumatic cardiac arrest patients.

Survivors were more likely to be treated at medical centers (40.8% vs 30.5%, *P* < .001) and hospitals equipped with a higher number of ICU beds. However, the distribution of hospital geographic areas was variable, as shown in Table 2; 48.0% of survivors and 42.9% of nonsurvivors attended hospitals located in metropolitan areas such as northern Taiwan.

**Table 2:**
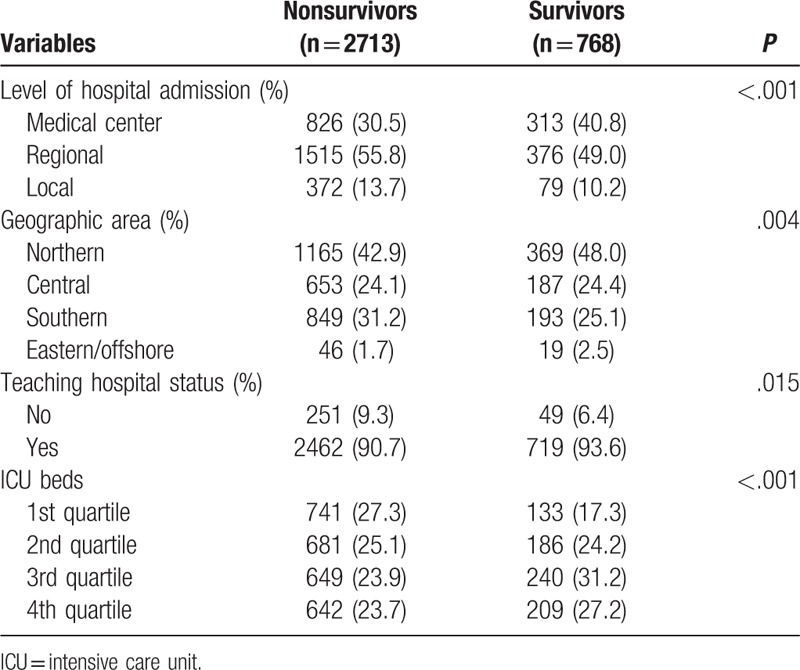
Features of healthcare systems where hospitalized traumatic cardiac arrest patients received treatment.

Table 3 shows the descriptive outcome of event characteristics. VF cardiac rhythm on admission was 20.1% and 4.5% in survivors and nonsurvivors, respectively. The survivor group had a higher proportion of transfers to another hospital than the nonsurvivor group (28.3% vs 14.8%, *P* < .001). In the pattern of injury, the leading causes of hospitalized TCA were traffic accidents, falls, and drowning/suffocation. Patients with survival to discharge were less likely to experience traffic accidents than those without survival to discharge (9.1% vs 18.9%, *P* < .001). Additionally, the survival conditions of the 2 groups were not significantly different between various periods of the calendar year.

**Table 3:**
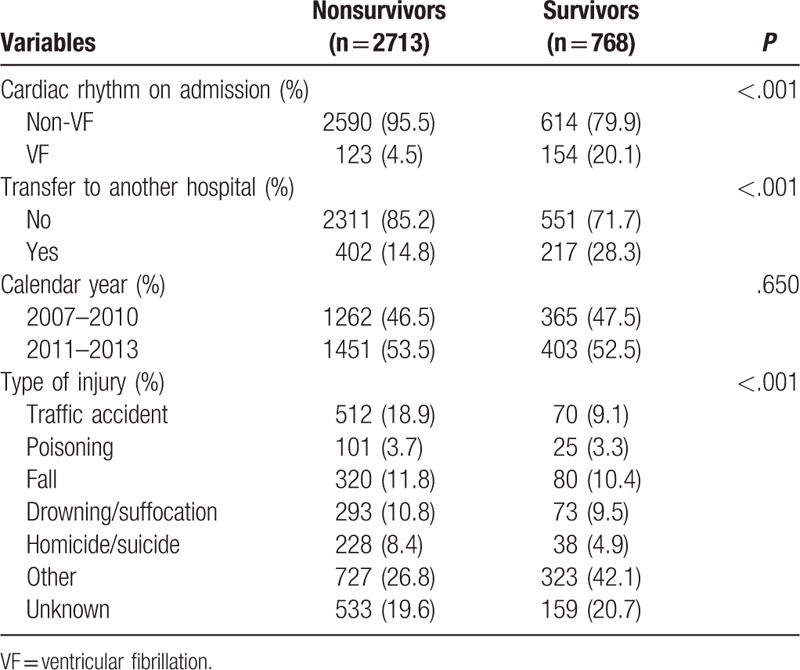
Characteristics of hospitalized traumatic cardiac arrest event parameters.

As shown in Table 4, multivariate logistic regression indicated a decreased OR by the number of organ failures (adjusted OR [aOR]: 0.82; 95% CI: 0.73–0.92) and southern geographic area (aOR: 0.79; 95% CI: 0.64–0.99). By contrast, patients treated by hospitals with a higher number of ICU beds had an increased probability of hospital discharge. As expected, VF was a dominant predictor of prognosis corresponding to non-VF cardiac rhythm on admission (aOR: 4.33; 95% CI: 3.29–5.70). Hospitalized TCA patients transferred to another hospital had a better chance of survival to discharge (aOR: 2.33; 95% CI: 1.90–2.85). Compared with traffic accidents, different injuries associated with survival to discharge were identified; the aOR (95% CI) was 1.89 (1.12–3.19) for poisoning, 1.63 (1.13–2.36) for falls, and 2.00 (1.36–2.92) for drowning/suffocation except for homicide/suicide.

**Table 4:**
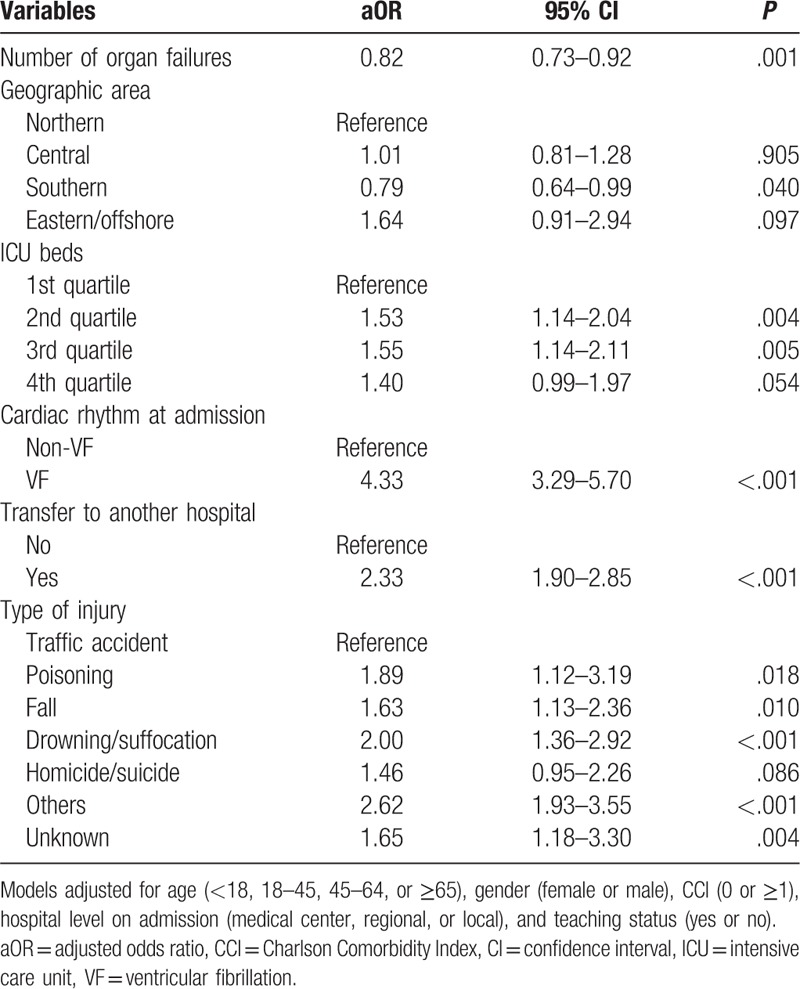
Survival analysis of hospitalized traumatic cardiac arrest patients by using multiple logistic regression with the enter model.

## 4 Discussion

With data obtained from a country-level database, we comprehensively analyzed various types of injuries and assessed the determinants of the survival of hospitalized TCA cases. Traffic accidents, falls, and drowning/suffocation contributed to approximately half of hospitalized TCA events among the Taiwanese population from 2007 to 2013. There was a relatively lower proportion of survival to discharge among hospitalized TCA cases caused by traffic accidents. After adjustment for covariates, the results showed that a lower number of organ failures, higher number of ICU beds, VF cardiac rhythm on admission, and transfers to another hospital increased survival to discharge.

A study by Huber-Wagner et al in Germany revealed that survival rates to discharge among severely injured patients who received external cardiac massage in the trauma room and emergency thoracotomy in the hospital were 11.5% and 13.0%, respectively.^[20]^ Recently, Beck et al^[21]^ reported that 15% of traumatic out-of-hospital cardiac arrest (OHCA) patients in Australia survived to discharge. Furthermore, several studies were conducted to survey the survival outcome of hospitalized TCA patients; these studies concluded that the rate of survival to discharge was approximately 16% to 23%.^[6,22]^ Thus, the survival-to-discharge rate of traumatic OHCA patients admitted to the hospital in prior studies was approximately 10% to 23%. In our study, hospitalized TCA patients had a survival rate of 22.1%, consistent with the findings of previous studies.

In a number of studies, the average age of traumatic OHCA patients, analyzed as a continuous variable, was lower than that of nontraumatic OHCA patients.^[6,21–24]^ Approximately 60% of OHCA patients suffering from trauma were under 65 years old, and males accounted for approximately 60% to 80% of cases.^[25–27]^ Most previous studies also noted that demographic indicators, such as age and gender, were associated with survival. However, our investigation detected no significant differences in survival outcome with those variables, similar to the findings of a Canadian study.^[26]^ There are 2 possible explanations for this disparity. The first is our inclusion criteria for TCA patients and survival to hospitalization after resuscitation. Patients with severe injuries who died at the scene or emergency department were not included in our study. Regarding the methods of the study conducted in Canada, Evans et al excluded TCA patients with obvious signs or injuries incompatible with life.^[26]^ The other explanation is that we included not only out-of-hospital TCA patients but also in-hospital TCA patients during hospitalization. Therefore, these differences probably shifted the demographic characteristics and further impacted the association between patients’ data and relevant independent variables.

Consistent with other reports, this study demonstrated that the presence of clinical symptoms and conditions such as organ failure preclude a favorable prognosis after hospital admission.^[16,25]^ The main goal of calculating the number of organ failures was to evaluate the collective impacts on survival and determine the collinearity between the clinical characteristics. A study by Chakravarthy et al found that the rate of survival to discharge was dramatically decreased among cardiac arrest patients with more than 1 comorbidity, similar to our finding that the survival rate decreased with an increase in the number of organ failures.^[28]^ We also applied the CCI to determine the effects of different types of existing clinical features and diseases on prognosis.^[17,18]^ The CCI had no significant adverse impact on survival in our study, but further studies are required to elucidate the influence of the CCI on survival conditions.

In the present study, there was interarea/hospital variability in survival outcomes. Based on the chain of survival, it is noteworthy that immediate cardiopulmonary resuscitation and appropriate prehospital medical care are important contributors to the outcome of cardiac arrest events. Cardiac arrests that occurred in rural and remote locations were not easily witnessed by bystanders and approached by emergency medical services, causing delayed hospital treatment. There is a reasonably lower rate of survival among patients in areas lacking medical resources. This lower survival rate could be due to varying levels of medical resources in different areas. In addition, observations in our audit revealed that a healthcare system feature, ICU bed number, was positively correlated with prognosis. This association has also been reported in previous studies and emphasized the importance of postcardiac arrest care in a monitored environment.^[15,16]^ The hospital level on admission was not associated with prognosis, but a higher number of ICU beds also often represent a more advanced level of hospital. In accordance with our conclusion, the effect of ICU bed number on hospitalized TCA survival appears to override the effect of hospital level on admission. Nevertheless, some results indicated variations in survival conditions that remained unexplained due to regional and hospital variations.^[21,29,30]^

Our results demonstrated that cardiac rhythm on admission was a remarkable predictor of the outcome. Consistent with several previous studies, cardiac arrest patients with VF/VT rhythm (shockable rhythm) had better survival than those without it.^[25,31]^ Wolbinski et al^[32]^ reported that OHCA patients with VF/VT presentations had much a higher survival rate than those without it (27% vs 1%). Because VF/VT rhythm was recorded in only 3% of total traumatic OHCA patients compared with 17.1% of medical OHCA patients, the survival rate of traumatic OHCA patients was significantly lower.^[6]^ Even though 11.4% of patients who achieved spontaneous circulation on admission presented with initial shockable rhythm, the proportion of shockable rhythm was elevated to 25% among the patients who survived to discharge.^[22]^ This phenomenon was consistent with our findings that hospitalized TCA cases with survival to discharge had a much higher proportion of VF rhythm than those without survival to discharge (19.0% vs 4.0%). The underlying mechanism of the higher rate of VF rhythm among hospitalized TCA cases with survival to discharge remains unclear. However, Georgescu et al reported that one-third of traumatic OHCA patients also presented with cardiovascular injuries or lesions, which is a reasonable explanation for our finding.^[23]^ It is very possible that cardiac problems increased the chances of VF/VT cardiac rhythm among hospitalized TCA patients. Consequently, VF/VT rhythm still plays an important role in improving the survival of different types of cardiac arrest cases in this study.

Multivariate logistic regression showed increased odds of survival to discharge among patients transferred to another hospital. In the present study, our survival rate does not refer to all cardiac arrest events due to trauma but only to hospitalized TCA. Patients with no bystander resuscitation and obvious signs of irreversible injury have higher chances of dying in the early stage of hospitalization. Therefore, it is less likely that those hospitalized TCA patients survive long enough to be transferred to another hospital. Specifically, transfer to another hospital is positively associated with an independent outcome due to systematic preselection of the cohort.

In line with our conclusion, previous studies noted that the leading TCA etiologies were traffic accidents and falls.^[6,21,22,24]^ Additionally, we reported the proportions of other injury types, such as drowning/suffocation, homicide/suicide, and poisoning. As described above, traffic accidents account for 40% to 60% of TCA cases. However, only approximately 20% of our study cases were attributed to traffic accidents. This difference occurred because most studies collected all TCA patients for analysis, and we included only hospitalized TCA patients in our study to enhance the importance of postcardiac arrest care. The much lower percentage of TCA cases caused by traffic accidents in this study seem to indicate a negative prognosis after cardiac arrest among traffic accident victims. In the multivariate analysis, compared with traffic accidents, other injury types were associated with a higher possibility of survival to discharge among hospitalized TCA cases. Our findings are similar to those of previous studies. Koller et al^[33]^ also reported that suspected drug overdose-related cardiac arrest had a positive association with survival. TCA caused by falls might have higher survival-to-discharge chances than TCA caused by traffic accidents.^[22,26,28]^ Lockey et al^[34]^ reported that TCA caused by drowning/suffocation tended to have increased odds of survival to discharge. In addition, our study provides further descriptions of borderline-nonsignificant elevated survival among hospitalized TCA patients due to homicide/suicide. According to a previous study, the majority of traffic accidents caused blunt TCA.^[34]^ In this study, we could not investigate the association between prognosis and injury mechanism because the data were unavailable. Initially, we illustrated different etiologies and compared the survival in different types of injury (Tables 1 and 2 in Supplemental Digital Content). Further studies are required to explore more aspects in order to clarify the detrimental effects of various injury mechanisms and etiologies on TCA outcome by subgroup analysis and find out how to improve the process of resuscitation.

## 5 Limitations and strengths

This study has inherent limitations. First, because the database comprises inpatient treatment records, only hospitalized TCA patients were included in this study. Therefore, patients who died at the scene and those who did not survive after resuscitation in emergency departments were excluded from the present study. Although some cases had missing information due to the characteristics of database, we focused on the survival rate of hospitalized TCA patients and emphasized the importance of postcardiac arrest care instead. Next, cardiac rhythm on admission recorded by ICD-9-CM codes does not account for patients whose presenting rhythm occurred at the onset of cardiac arrest due to characteristics of database. It is likely that we underestimated the proportion of VF among cardiac rhythms, thereby weakening its effect on survival condition. Third, external injury codes were absent for one-fifth of patients in our study. This issue might limit our ability to investigate the effects of injury patterns on the rate of survival to discharge. Despite the aforementioned weaknesses, we also determined the parameters associated with survival conditions and described the prognosis of different injury types compared with that of traffic accidents. In addition, the large population of this study diluted potential bias and confounders; thus, our results are more generalizable. Due to the small sample size, we classified burn injury patients into another group. Of 15 patients, only 1 ultimately survived to discharge (data not shown) in this study and could result in mild underestimation of survival probability in this group. Finally, based on our literature review, some TCA patients suffer from unfavorable neurological sequelae.^[6,7,26]^ However, the only outcome variable of this study was survival to hospital discharge. Therefore, the neurologic function of patients discharged to their homes or transferred to a long-term care center is unknown and could actually limit the extent of our conclusions. In addition, we had no detailed injury and treatment information (e.g., Injury Severity Score,^[23]^ Abbreviated Injury Scale,^[35]^ medication used before arrival) from NHIRD, and this issue may lead to residual confounders. Thus, more research is warranted to collect more data and evaluate this issue from retrospective medical record review.

## 6 Conclusion

This nationwide study concluded that hospitalized TCA patients with multiple organ failure may be less likely to be discharged from the hospital. The presence of VF rhythm increased the odds of survival to discharge. In the phase of postcardiac arrest care, 2 hospital variables, the number of ICU beds and transfer to another hospital, were positively correlated with survival. Our findings emphasized that hospitalized TCA events attributed to traffic accidents have a much worse influence on the main outcome.

## Author contributions

**Conceptualization:** Chung-Yu Lai.

**Data curation:** Chung-Yu Lai, Fu-Huang Lin.

**Formal analysis:** Chung-Yu Lai, Fu-Huang Lin, Chi-Hsiang Chung.

**Methodology:** Chung-Yu Lai, Shih-Hung Tsai, Chih-Hung Ku.

**Project administration:** Fu-Huang Lin, Chi-Hsiang Chung.

**Resources:** Wu-Chien Chien.

**Software:** Chi-Hsiang Chung.

**Supervision:** Shih-Hung Tsai, Chi-Ming Chu.

**Validation:** Shih-Hung Tsai, Hsin Chu, Chih-Hung Ku, Chun-Hsien Wu, Chi-Ming Chu.

**Visualization:** Chun-Hsien Wu.

**Writing – original draft:** Chung-Yu Lai.

**Writing – review and editing:** Shih-Hung Tsai, Hsin Chu, Chih-Hung Ku, Wu-Chien Chien, Ching-Tsan Tsai, Huan-Ming Hsu, Chi-Ming Chu.

## Supplementary Material

Supplemental Digital Content
