# Supplementary material for: Survival rate variation among different types of hospitalized traumatic cardiac arrest: A retrospective and nationwide study
Source: Medicine (Baltimore). 2018 Jul 13;97(28):e11480. doi: 10.1097/MD.0000000000011480 (PMC6076037; doi:10.1097/MD.0000000000011480)
Supplement: Supplemental Digital Content [file medi-97-e11480-s001.docx]

| **Supplemental table 1. Characteristics of different etiologies among hospitalized traumatic cardiac arrest patients.** | | | | |
| --- | --- | --- | --- | --- |
| **Variables** |  | **Non-survivors**  **(n=2713)** | **Survivors**  **(n=768)** | ***P*** |
| Head injury |  |  |  | <0.001 |
| No |  | 1995 (73.5) | 658 (85.7) |  |
| Yes |  | 718 (26.5) | 110 (14.3) |  |
| Neck injury |  |  |  | 0.096 |
| No |  | 2583 (95.2) | 742 (96.6) |  |
| Yes |  | 130 (4.8) | 26 (3.4) |  |
| Trunk injury |  |  |  | <0.001 |
| No |  | 2303 (84.9) | 713 (92.8) |  |
| Yes |  | 410 (15.1) | 55 (7.2) |  |
| Upper limb injury |  |  |  | 0.089 |
| No |  | 2633 (97.1) | 754 (98.2) |  |
| Yes |  | 80 (2.9) | 14 (1.8) |  |
| Lower limb injury |  |  |  | 0.009 |
| No |  | 2535 (93.4) | 737 (96.0) |  |
| Yes |  | 178 (6.6) | 31 (4.0) |  |

| **Supplemental table 2. Survival analysis associated with etiologies among hospitalized traumatic cardiac arrest patients by using multiple logistic regression with the enter model stratified by different types of injury.** | | | | | | | | | | |
| --- | --- | --- | --- | --- | --- | --- | --- | --- | --- | --- |
| **Variables** | **Traffic accident** | | **Poisoning** | | **Fall** | | **Drowning/Suffocation** | | **Homicide/Suicide** | |
|  | **aOR(95%)** | ***P*** | **aOR(95%)** | ***P*** | **aOR(95%)** | ***P*** | **aOR(95%)** | ***P*** | **aOR(95%)** | ***P*** |
| Head Injury |  |  |  |  |  |  |  |  |  |  |
| No | Reference |  | Reference |  | Reference |  | Reference |  | - |  |
| Yes | 0.39(0.23-0.68) | 0.001 | 4.17(0.25-69.03) | 0.319 | 0.64(0.38-1.07) | 0.087 | 3.90(0.49-31.27) | 0.200 |  |  |
| Neck Injury |  |  |  |  |  |  |  |  |  |  |
| No | Reference |  | - |  | Reference |  | - |  | Reference |  |
| Yes | 1.27(0.63-2.57) | 0.511 |  |  | 0.83(0.30-2.32) | 0.725 |  |  | 0.99(0.28-3.52) | 0.982 |
| Trunk Injury |  |  |  |  |  |  |  |  |  |  |
| No | Reference |  | - |  | Reference |  | Reference |  | - |  |
| Yes | 0.53(0.31-0.93) | 0.026 |  |  | 0.61(0.31-1.22) | 0.162 | 1.23(0.09-17.74) | 0.879 |  |  |
| Upper limb injury |  |  |  |  |  |  |  |  |  |  |
| No | Reference |  | - |  | Reference |  | - |  | - |  |
| Yes | 1.52(0.66-3.49) | 0.322 |  |  | 0.31(0.04-2.48) | 0.271 |  |  |  |  |
| Lower limb injury |  |  |  |  |  |  |  |  |  |  |
| No | Reference |  | - |  | Reference |  | - |  | Reference |  |
| Yes | 1.36(0.67-2.78) | 0.400 |  |  | 0.27(0.12-0.65) | 0.003 |  |  | 6.24(0.85-45.86) | 0.072 |
| aOR = adjusted odds ratio; CI = confidence interval. | | | | | | | | | | |
